# The Pandemic Lens: Focusing across Time Scales for Local-Global Sustainability

**DOI:** 10.1016/j.patter.2020.100147

**Published:** 2020-11-13

**Authors:** Paul Arthur Berkman

**Affiliations:** 1 United Nations Institute for Training and Research (UNITAR), 7 bis, Avenue de la Paix, CH-1202 Geneva 2, Switzerland; 2 Program on Negotiation, Harvard Law School, Harvard University, Pound Hall 501, 1563 Massachusetts Avenue, Cambridge, MA 02138, USA; 3 Science Diplomacy Center, Fletcher School of Law and Diplomacy, Tufts University, 160 Packard Avenue, Medford, MA 02155, USA

## Abstract

Humanity faces a series of challenges over a range of timescales from minutes to centuries that are relevant to our sustainable development as a globally interconnected civilization. Our common survival at local-global levels depends on being able to understand the urgencies of exponential change across these timescales. The “Pandemic Lens” introduced by the COVID-19 pandemic gives us perspective to operate with informed short-term to long-term decision making *for the benefit of all on Earth across generations.*

## Main Text

This essay is written from the perspective of a parent, recognizing that children and even young adults today will be alive in the 22^nd^ century. For those of you who will travel across the 21^st^ century, consider basic lessons of the COVID-19 pandemic across your lifetime with relevance at local-global scales across generations. A starting point in this journey is to appreciate we are living in a globally interconnected civilization, as revealed by our global pandemic. We now have a common interest in survival on a planetary scale with the spread of the coronavirus, providing a lens for humankind to focus on the urgencies of exponential change across a wide range of embedded timescales. The “Pandemic Lens” introduces the option (without advocacy) for all of us to evolve with informed decision making as part of lifelong learning, operating across a “continuum of urgencies,” short-term to long-term, *for the benefit of all on Earth across generations.*^1^

### Next Generations with Our Globally Interconnected Civilization

**Figure 1:**
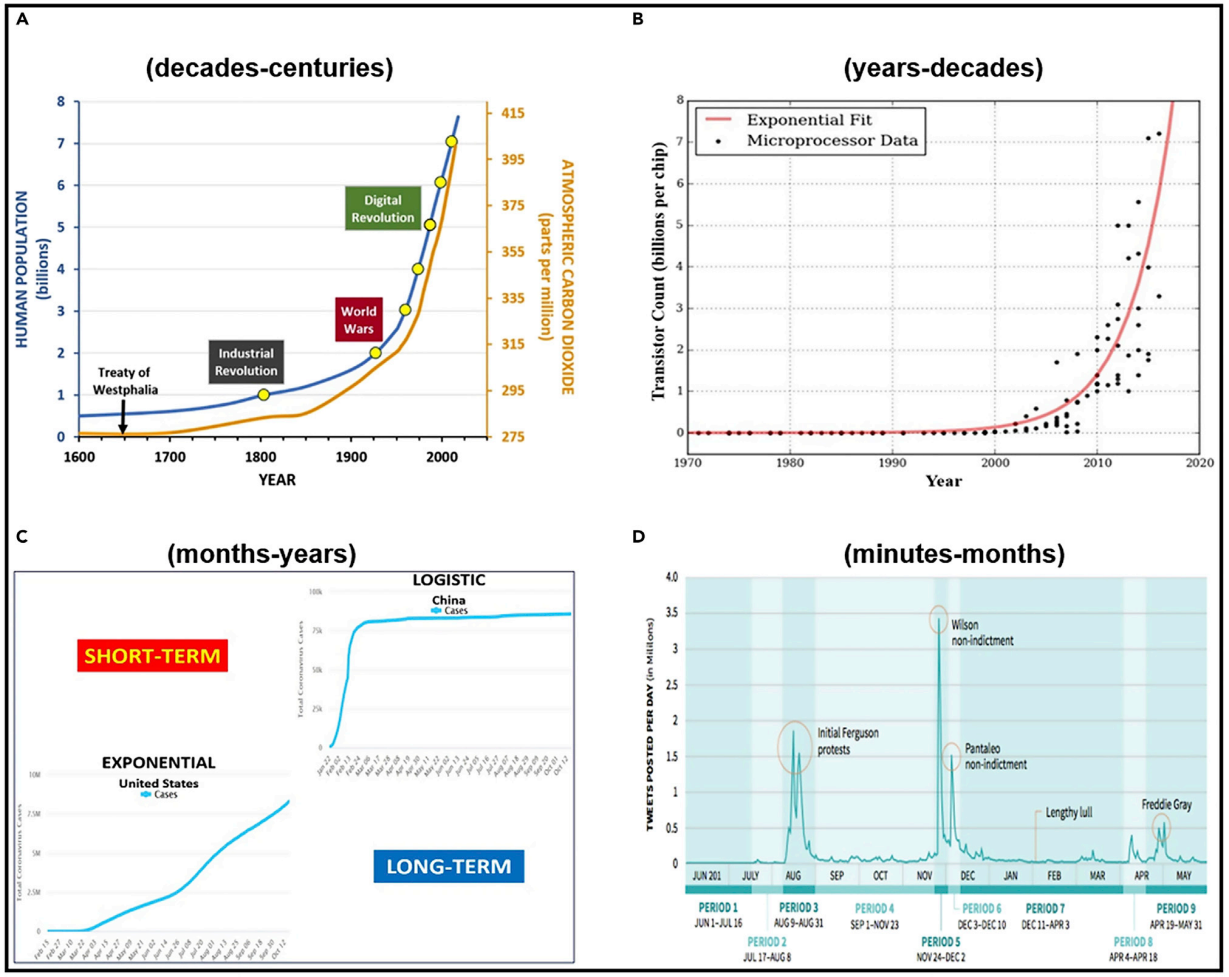
Globally Interconnected Civilization Time Scales Globally interconnected civilization timescales revealed by exponential changes with: (A) Climate and human-population change over decades to centuries,^1^ (B) high-technology change over years to decades illustrated by Moore’s Law with transistors on a chip, (C) global pandemic change over months to years with COVID-19 cases accelerating across the Earth, illustrated by the United States (scale of 10,000,000–10^7^) in contrast to China (scale of 100,000–10^5^) through October 12, 2020, as recorded by *Worldometer*, and (D) social-media change over minutes to months in relation to specific events, illustrated by 2014–2015 tweets about “Black Lives Matter,” posted per day (in millions), as reported in *Mother Jones* on March 13, 2016.

The unambiguous reality of Earth’s great civilizations throughout history is that we are now globally interconnected unlike any time in the past (Figure 1). This fact is demonstrated simply by the concept of “world wars”, which happened for the first time in the history of humankind only in the last century, noting we are in the grip of the COVID 19 pandemic, the *“most challenging crisis we have faced since the Second World War.”*^2^ For perspective, the oldest continuous annual calendars produced by humans record nearly 6000 years—with the past few centuries as in a lifespan of sixty centuries—demonstrating with Earth-system impacts, we are still in our infancy to operate on a planetary scale.

Fundamentally, the past few centuries have seen an accelerating increase in global human population size, which is orders of magnitude larger than at the dawn of the nation-state with the *Treaty of Westphalia* in 1648. The first (2^0^) billion people were alive around 1800 at the start of the Industrial Revolution, when fossil-fuel burning started to significantly impact our home planet and climate. Two (2^1^) billion people were alive just after the first world war, as the Spanish Flu took hold, when we were faced with our last global pandemic, which killed nearly fifty million people.^3^ We entered the Digital Revolution with four (2^2^) billion people living on Earth, and are now progressing to eight (2^3^) billion people on Earth this decade. Throughout the exponential trajectory of human population growth—across decades to centuries—there have been parallel impacts to our planet’s climate with increasing carbon dioxide mixed into the atmosphere around the Earth (Figure 1A).

The challenge for our globally interconnected civilization is to mature with knowledge co-production among allies and adversaries alike, building common interests at local-global levels for sustainable development. With climate—being aware of global urgencies across decades to centuries (Figure 1A)—we are still early in our shared journey that started with the 1992 *United Nations Framework Convention on Climate Change* (UNFCC), which introduced a globally inclusive process with ongoing Conferences of the Parties (COP). Synergistic outcomes of the UNFCC are illustrated by global research with the Intergovernmental Panel on Climate Change evolving into global action with the 2015 Paris Accord, which was COP21. The geography of the climate challenge underscores the ability to integrate the capacities of humankind, operating in harmony across the whole jurisdictional spectrum with its subnational-national-international levels, now and into the future, on a planetary scale.^4^

### Informed Decision Making across a Continuum of Urgencies

What we seek as a globally interconnected civilization is the perspective to make decisions that operate over decades to centuries, anchored to the realities of what we currently are facing. Where does decade-century perspective come from and how we can derive insights ourselves that are helpful at local-global levels? A practical answer is to look at shorter timescales with analogous considerations that are easier to touch and see than across decades to centuries.

For example, across years to decades there is exponential change with high technologies, as symbolized with the number of transistors on a computer chip, progressing from thousands (10^3^) in the late 1960’s to billions (10^9^) in the past decade (Figure 1B). An outcome is the acceleration of questions about the opportunities and challenges created by high-technology products, services, and institutions that touch our lives continuously, connecting billions of people across the Earth.

These connections between humans on Earth can be seen with data and initiated with holistic (international, interdisciplinary, and inclusive) inquiry without boundaries. The path between questions and data involves the natural sciences and social sciences as well as Indigenous knowledge, all of which characterize patterns, trends, and processes (albeit with different methods) that become the bases for decisions. Such holistic research involves science as the *“*study of change*”* to identify as well as address questions of common concern. The outcome is common-interest building to deliver informed decisions,^4^ operating across a “continuum of urgencies”^5^ as a holistic process that starts with questions among allies and adversaries alike.

**Figure 2:**
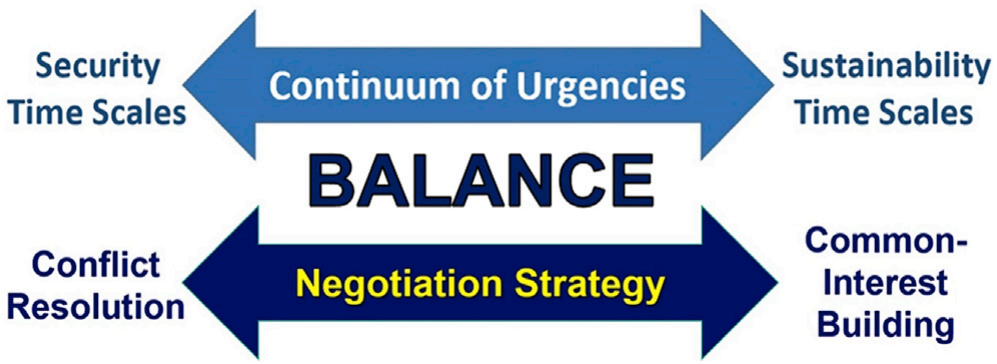
Informed Decision Making Informed decision making as a scalable proposition, illustrated for peoples, nations, and our world from security timescales (mitigating risks of political, economic, cultural, and environmental instabilities that are immediate) to sustainability timescales (balancing economic prosperity, environmental protection, and societal well-being across generations) with “conflict resolution” and “common-interest building” as negotiation strategies to achieve balance with issues, impacts, and resources at local-global levels.^7^

With informed decision making as the apex goal, the path to both promote cooperation and prevent conflict recognizes that the journey is determined by the starting point. Cooperation emerges from common-interest building and holistic integration, as with the two superpower adversaries throughout the Cold War in Antarctica and outer space.^6^ Alternatively, conflict resolution is polarizing from the start, unbalanced by short-term considerations. The challenge is to operate short-term to long-term at all levels in our world, building beyond the predominating negotiation strategy of conflict resolution (Figure 2).

Informed decision making extends beyond research and into action,^1^^,^^4^^,^^7^ recognizing that data to answer questions are different than evidence for decisions, both in purpose and design (i.e., data does not equal evidence). Evidence integrates data in view of the institutions that make decisions about governance mechanisms (laws, agreements, and policies as well as regulatory strategies, including insurance, at diverse jurisdictional levels) as well as built infrastructure (fixed, mobile, and other assets, including communication, observation, information, and other systems that require technology plus investment).

However, evidence only compels the decisionmakers to act, emphasizing the importance of options (without advocacy), which can be used or ignored explicitly, to avoid the polarization of politics that emerge with agendas in view of the decision making institutions. With informed decisions operating across time (Figure 2), coupling between governance mechanisms and built infrastructure is possible to manage, as envisioned by the seventeen United Nations Sustainable Development Goals^8^ with their targets and indicators as a gift for humanity.

### Before-through-After Global Inflection Points

Poignantly, we are living in a world of exponential change over months to years with our global pandemic (Figure 2C). In the United States—where the first (10^0^) reported COVID-19 mortality was on March 1, 2020—impacts of the severe acute respiratory syndrome coronavirus 2 (SARS-CoV-2) have accelerated through the nation from 10 (10^1^) to 100 (10^2^), 1,000 (10^3^), 10,000 (10^4^), and beyond 100,000 (10^5^) deaths by May 27, 2020.^9^ The reality check is that more than 99% of the human population of nearly 8 billion people (Figure 1A) remains to be attacked by COVID-19. Once again, unlike any period since the second world war, we all share a common interest in survival, hoping for a vaccine with global distribution that will end this plague of our time.

Juxtaposed with the exponential growth of our global pandemic is logistic change, as illustrated by the arrested spread of COVID-19 in China, the epicenter of our global pandemic months ago (Figure 1A). The sigmoid shape of logistic change is exactly what is anticipated when humankind effectively addresses carbon dioxide in the global atmosphere, for example.

An already important outcome of our global pandemic is the lens that we can use to see that humankind can manage exponential change, as in China and in many other countries where there is informed decision making short-term to long-term (Figure 1C). The unfortunate alternative is uninformed decision making that perpetuates exponential change by focusing on short-term considerations only, as revealed in the United States at the “Twitter Timescale” or “Political Timescale” of social media, reacting over minutes to months (Figure 1D). The severity of uninformed decisions touches us all when security and sustainability timescales (Figure 2) are disconnected.

**Figure 3:**
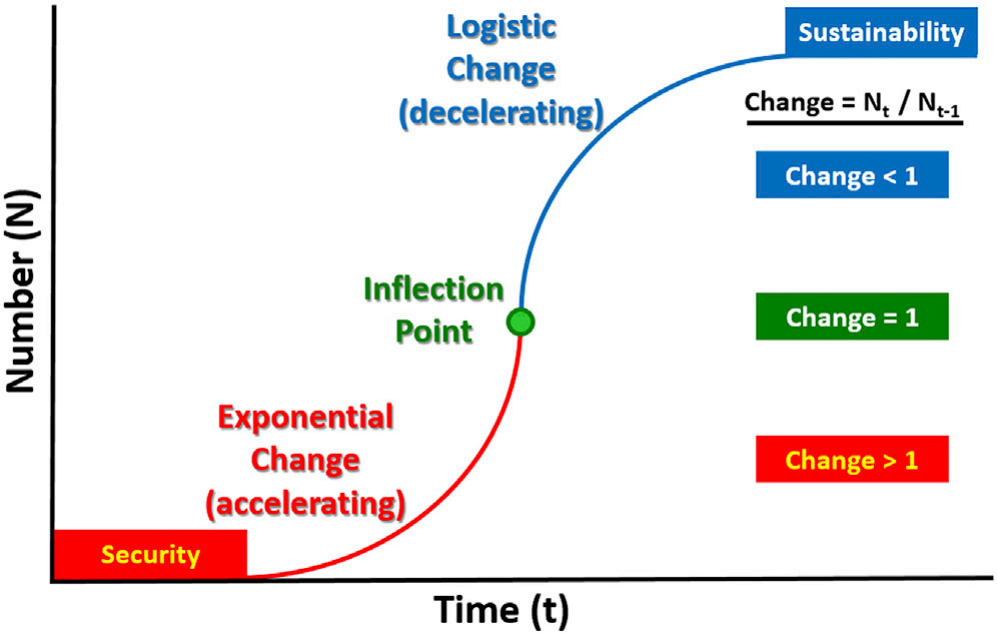
“Pandemic Lens” for Sustainability “Pandemic lens” for sustainability, highlighting exponential change across an inflection point toward logistic (S-shaped, sigmoid) change, as described by numbers (N) changing per unit of time (t). Informed decisions operate across a “continuum of urgencies” (Figure 2)—short-term to long-term before-through-after inflection points—with scalability across embedded timescales of our globally interconnected civilization (Figures 1A–1D). Adapted from Berkman, 2020.^7^

What are the lessons from the COVID-19 pandemic that apply to longer timescales (Figures 1A–1B) across a “continuum of urgencies” with our sustainable development (Figure 2)? Sometime between now and infection of the entire human population, the exponential destruction of lives and livelihoods will decelerate across the Earth—which is a source of hope—and we will enter the long tail of logistic change (Figure 1C). When this global inflection point will arrive is unknown, but it will arrive, integrating the diverse regional and national responses on a planetary scale (Figure 3).

The “Pandemic Lens” (Figure 3) is a mental model to operate across multiple timescales before through after inflection points. Like a telescope or microscope, the “Pandemic Lens” is available for all to consider short-term and long-term changes, impacts, and responses across a “continuum of urgencies” (Figure 2). Over months to years, years to decades, and decades to centuries (Figures 1A–1D), there is opportunity to construct informed decisions that optimize the available information that is practicably infinite and instantaneous in our digital era.

With our globally interconnected civilization (Figures 1A–1D), the relevance of operating across a “continuum of urgencies” (Figure 2) has precedent. The Bretton Woods and San Francisco conferences before the end of World War II enabled nations to envision a “new world order” based on their common interests^10^ and to put into place built infrastructure coupled with governance mechanisms after the inflection point in August 1945, when the second world war ended across the Earth.^7^ We have the same opportunity now before the global inflection point with the COVID-19 pandemic, triangulating education, research, and leadership to reveal options for an era of informed decision making with lifelong learning for those who will journey across the 21^st^ century. With hope and inspiration for humanity, the “Pandemic Lens” (Figure 3) illustrates the path for us to evolve as an interconnected civilization with informed decision making at local-global levels, focusing with compassion short-term to long-term *for the benefit of all on Earth across generations.*
